# Structural, biological and pharmaceutical importance of antibiotic agent chloramphenicol

**DOI:** 10.1016/j.heliyon.2020.e03433

**Published:** 2020-03-04

**Authors:** A. Sathya, T. Prabhu, S. Ramalingam

**Affiliations:** a Department of Physics, A.V.C. College, Mayiladuthurai, Tamilnadu, India; b Affiliated to Bharathidasan University, Tiruchirappalli, Tamilnadu, India

**Keywords:** Theoretical chemistry, Pharmaceutical chemistry, Chloramphenicol, α-hydroxyl group, CT complex, Hyperactive polarization, Biological properties, Hyperactive polarizability

## Abstract

The vibrational, magnetic resonance and electronic spectral techniques are used to evaluate structural activity associated physico-chemical properties. The biological affinity and drug importance was validated by calculating biological parameters using HyperChem. Mulliken charge assignment for restoring chemical potential for generating drug potential in the molecular site was mapped and analyzed. The vibrational spectral pattern was estimated by identifying active and inactive bands and hindrance of vibrational activity of Acetamide group was monitored and thereby drug malfunction was tested. The chemical reaction pathway around the core carbons of chain and ring was keenly noted and the cause of chemical potential for the inducement of drug mechanism was reported. The stimulation of chemical mechanism for antibiotic activity was addressed by suitable evidence and further improvement for enhancing activity was made. The electronic HOMO and LUMO interaction over different molecular entities are discussed to expose accompany of drug mechanical transitions. The CT complex was recognized to be C=N and C=C bonds and operating drug mechanism was monitored. The unwanted drug property induced by perplexes of charge depletion on α-hydroxyl group was assessed from MEP map. The hyperactive polarization energy of 266.18 X10^−33^ esu and 327 X10^−33^ esu of present compound is causing biological activity in good order. The uncontrolled breathing region of Acetamide group was clarified in VCD profile and this is main cause to produce toxicity in drug process.

## 1 Introduction

The Chloramphenicol also called as D-(-)-2,2-dichloro-N-(β-hydroxy-α-(hydroxymethyl)-p-nitrophenylethyl)Acetamide is bacteriostatic activity enabled antibiotic agent and it makes binding interaction with 50S ribosomal subunit. Thereby it put off the transformation of amino acids to the growing peptide chain and obstructing peptide bond configuration [1]. Then, the peptide bond was saturated suddenly and the peptide bond configuration altered in such a way that, bacterial protein synthesis is stopped and hinders the bacterial cell production. The chloramphenicol is chlorine aided organic complex called di-Chloro-Acetamide in which nitrobenzene ring is injected with an amide bond and two alcohol species [2]. Due to such combinations, it is acted as an antimicrobial mediator and an antibacterial medicine. Normally the chloramphenicol in the form of injection may be used to treat and prevent hazardous illnesses due to severe tularemia and plague. It is primarily used for typhoid and fever and also to treat bacterial eye infections. Chloramphenicol is recommendation for antibiotic to administrate in to vein for curing severe infections and systemic infections [3] since the rapid renovation of amino acid combination in peptide chain is terminated.

It is widely used as topical ointment applied consistently to suture pattern lines, skin grafts and mainly used to prevent infections on the face and around the eyes that wounded in accident. Due to the good bactericidal capability, it is widely used in disease control in animal husbandry and aquaculture form [4]. However, the use of chloramphenicol is associated with side effects, such as aplastic anemia, the structure are needed to be altered to reduce toxic level as well as side effects. The unwanted drug process is usually induce toxicity in the metabolic process and such active source of molecule present in the compound to be identified and replaced to avoid such unusual toxicity. Here, the toxic species is identified and replaced by suitable ligand groups that will not affect the optimized drug process. In this work, the toxicity is identified and counterpart ligand group has been added in suitable place in molecular site and the result is analyzed using spectroscopic and biological test parameters.

## 2 Experimental methodology

The present drug compound; chloramphenicol was purchased from Good Scents Company, USA, and it has been found to be high-quality and spectroscopic grade used for recording the spectra.

• The IR instrument with FT device (Bruker IFS 66V) was used to record spectra at high spectral resolution using adequate scanning speed.
• The FT-Raman spectral sequence was also recorded using Bruker spectrometer adopted with FT-Raman module.
• The ^1^H NMR and ^13^C NMR spectra were recorded using 300 MHz and 75 MHz NMR spectrometer with high magnetic gradient.
• The UV-Vis spectrum was recorded in solid phase in the region of 100–800 nm, with the scanning interval of 10 nm, using the UV-1750 series instrument.

## 3 Computational methods

Different type of theoretical calculations was performed for different parameters with respect to the molecular specification. The confined structure was optimized and its parameters were computed using GAUSSIAN 16 D .01 version software with the help of IMAC computer. The mulliken charge arrangement was captured as in the Gauss view and its coefficient was measured by B3LYP/6-311++G(d,p). The vibrational frequency pattern was recorded using hybrid DFT theory with B3LYP and B3PW91 theory at 6-311++G(d,p) mode. The ^1^H and ^13^C NMR isotropic and anisotropic form of chemical shifts were predicted using GIAO method by suitable I-PCM model at B3LYP/6-311++G(2d,p) mode. The entire Lipinski parameters calculations were performed using HyperChem software 8.0.6 version and were validated by Osiris program. The MEP charge depletion zones are identified and described in Figure. The regular and total linear polarizability and first order hyper active polarizability in different internal coordinates were calculated on B3LYP [6-311++G(d,p)] method. The enantiometric VCD spectral pattern was sketched and the chiral characteristics of compound were predicted.

## 4 Results and discussion

### 4.1 Molecular geometry distortion analysis

The molecular geometry is usually revealed details of physical parameters on par with compositional parts of molecule by which the physical properties can be interpreted. Here, the base compound was nitro benzene as well as Acetamide in which all other groups are purposively injected in order to induce antibiotic properties. As per the literature [5], as the nitrobenzene used as drug, the Acetamide and their α-hydroxymethyl and β-hydroxyl groups' combinations created aggressive antibiotic activity. According to the standard bond length of benzene ring, the CC bond length is in the region of 1.390–1.395Å [6]. Since the nitro group and chain were adopted with benzene, some of the bond lengths were altered simultaneously, the corresponding location elements (C20-C22 = 1.399Å, C22-C25 = 1.389Å and C20-C21 = 1.398Å) were stained positively. This influences internuclear disturbance and such that, the electron domain was rather shifted which ensured the blending of physical property of ring and chain. By the chloro substitution on C11 and carbonyl ligand on C10, the bond length of C10-C11 was found to be stretched more than other CC in the chain, which was to be 1.545Å. The hydroxymethyl on C9 and CHOH on C7, the internuclear distance between C7-C9 was found to be 1.526Å. Similarly, the bond length of C7-C8 was observed to be 1.557Å on same chain. Thus, bond length changes were addressed on each and every group that represents the simultaneous blending of chemical property of all ligand groups was observed to be antibiotic and antifungal. The similar effect was addressed in some of ligand groups for coherent characteristics of molecule [7].

**Figure 1:**
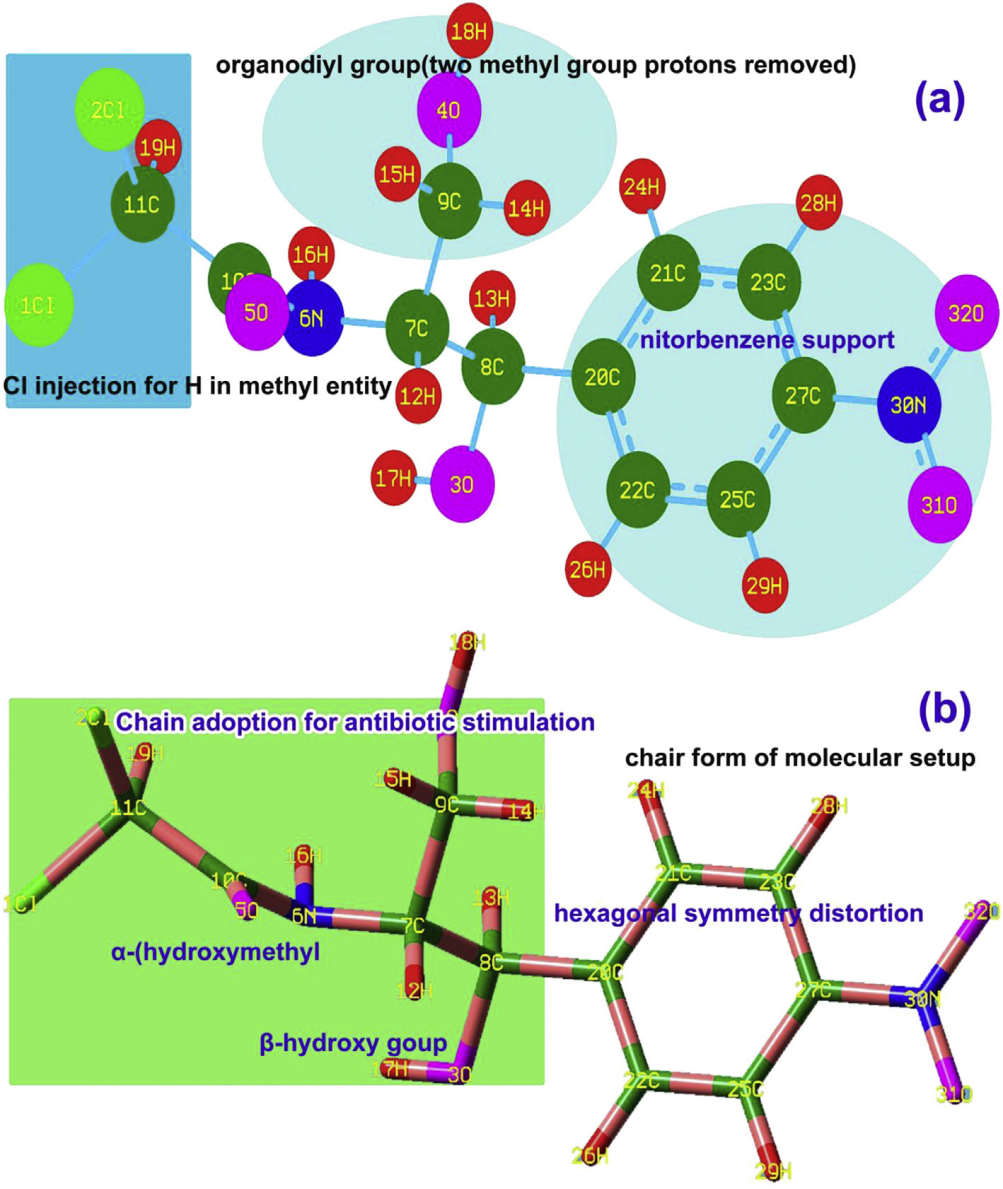
Molecular structure (a) Bond type (b) Tube form of Chloramphenicol.

As in the Table 1 of optimized parameter, the bond length and bond angles of Acetamide and its associated groups were altered at very high rate and it was unusual. The bond angles were distorted in ambiguity form which denoted that, the substitutional groups were not attached and grouped randomly. This atmosphere in molecular site should produce strange chemical property in the compound and the toxicity is induced above the expected limit. Here, in this compound the same effect was induced and monitored. The bond parameter diagram is shown in Figure 1. In which the change of bond parameters were clearly shown in substitutional group vice.

**Table 1:** Optimized geometrical parameters of Chloramphenicol.

Geometrical Parameters	Methods		
	HF	B3LYP	B3PW91
	6-311++G (d,p)	6-311++G (d,p)	6-311++G (d,p)
Bond length (Å)			
Cl 1-C11	1.782	1.808	1.794
Cl2-C11	1.758	1.777	1.763
O3-C8	1.398	1.421	1.412
O3-H17	0.941	0.964	0.963
O4-C9	1.408	1.433	1.425
O4-H18	0.940	0.961	0.960
O5-C10	1.184	1.209	1.207
N6-C7	1.457	1.463	1.456
N6-C10	1.353	1.367	1.363
N6-H16	0.995	1.011	1.011
C7-C8	1.545	1.557	1.552
C7-C9	1.522	1.526	1.521
C7-H12	1.080	1.091	1.093
C8-H13	1.085	1.096	1.098
C8-C20	1.515	1.514	1.509
C9-H14	1.084	1.094	1.095
C9-H15	1.086	1.095	1.096
C10-C11	1.538	1.545	1.542
C11-H19	1.075	1.086	1.088
C20-C21	1.388	1.398	1.396
C20-C22	1.390	1.399	1.397
C21-C23	1.383	1.390	1.388
C21-H24	1.075	1.084	1.085
C22-C25	1.381	1.389	1.387
C22-H26	1.073	1.082	1.083
C23-C27	1.379	1.390	1.388
C23-H28	1.071	1.081	1.082
C25-C27	1.382	1.391	1.389
C25-H29	1.071	1.081	1.082
C27-N30	1.465	1.479	1.473
N30-O31	1.187	1.224	1.218
N30-O32	1.188	1.225	1.219
Bond angle (°)			
C8-O3-H17	110.0	108.5	108.0
C9-O4-H18	110.6	109.4	109.2
C7-N6-C10	122.9	122.8	122.7
C7-N6-H16	116.1	115.8	115.9
C10-N6-H16	118.8	119.4	119.5
N6-C7-C8	108.8	109.2	109.1
N6-C7-C9	109.3	109.3	109.4
N6-C7-H12	107.0	106.6	106.6
C8-C7-C9	114.4	114.1	114.0
C8-C7-H12	108.4	108.3	108.3
C9-C7-H12	108.7	109.1	109.2
O3-C8-C7	110.0	110.1	110.1
O3-C8-H13	110.1	110.3	110.5
O3-C8-C20	107.5	107.7	108.0
C7-C8-H13	108.5	108.2	108.1
C7-C8-C20	112.4	111.9	111.5
H13-C8-C20	108.3	108.7	108.6
O4-C9-C7	107.9	107.4	107.4
O4-C9-H14	110.9	111.0	111.2
O4-C9-H15	110.6	110.7	110.9
C7-C9-H14	110.3	110.1	109.9
C7-C9-H15	108.6	108.9	108.7
H14-C9-H15	108.4	108.7	108.6
O5-C10-N6	125.1	124.9	125.0
O5-C10-C11	122.2	122.7	122.8
N6-C10-C11	112.6	112.3	112.2
Cl 1-C11-Cl 2	111.6	111.9	112.0
Cl 1-C11-C10	108.3	108.2	108.1
Cl 1-C11-H19	106.4	105.9	106.1
Cl 2-C11-C10	111.7	111.2	111.1
Cl 2-C11-H19	107.2	107.1	107.3
C10-C11-H19	111.5	112.4	112.2
C8-C20-C21	120.3	120.4	120.4
C8-C20-C22	120.3	120.3	120.2
C21-C20-C22	119.3	119.3	119.4
C20-C21-C23	120.8	120.8	120.8
C20-C21-H24	119.9	119.7	119.6
C23-C21-H24	119.3	119.5	119.6
C20-C22-C25	120.6	120.6	120.5
C20-C22-H26	119.8	119.5	119.4
C25-C22-H26	119.6	120.0	120.1
C21-C23-C27	118.5	118.5	118.5
C21-C23-H28	121.3	121.7	121.9
C27-C23-H28	120.2	119.7	119.6
C22-C25-C27	118.7	118.8	118.8
C22-2C5-H29	121.2	121.6	121.7
C27-C25-H29	120.1	119.7	119.6
C23-C27-C25	122.0	121.9	122.0
C23-C27-N30	118.9	118.9	118.9
C25-C27-N30	119.1	119.1	119.1
C27-N30-O31	117.6	117.7	117.6
C27-N30-O32	117.6	117.6	117.6
O31-N30-O32	124.7	124.7	124.8
Dihedral angles (°)			
H17-O3-C8-C7	55.01	51.18	49.08
H17-O3-C8-H13	-64.52	-68.13	-70.30
H17-O3-C8-C20	177.67	173.47	171.07
H18-O4-C9-C7	176.21	173.79	173.70
H18-O4-C9-H14	-62.82	-65.79	-65.98
H18-O4-C9-H15	57.56	55.05	54.99
C10-N6-C7-C8	124.18	120.46	120.79
C10-N6-C7-C9	-110.26	-114.13	-113.86
C10-N6-C7-H12	7.31	3.65	4.06
H16-N6-C7-C8	-72.89	-75.50	-75.21
H16-N6-C7-C9	52.66	49.91	50.14
H16-N6-C7-H12	170.23	167.69	168.06
C7-N6-C10-O5	-5.20	-4.57	-4.67
C7-N6-C10-C11	177.19	178.05	177.92
H16-N6-C10-O5	-167.70	-168.07	-168.12
H16-N6-C10-C11	14.69	14.55	14.47
N6-C7-C8-O3	-59.31	-57.00	-56.50
N6-C7-C8-H13	61.18	63.56	64.30
N6-C7-C8-C20	-179.02	-176.75	-176.39
C9-C7-C8-O3	178.16	-179.60	-179.09
C9-C7-C8-H13	-61.34	-59.03	-58.29
C9-C7-C8-C20	58.45	60.65	61.02
H12-C7-C8-O3	56.67	58.73	59.12
H12-C7-C8-H13	177.17	179.29	179.93
H12-C7-C8-C20	-63.03	-61.02	-60.77
N6-C7-C9-O4	-59.50	-58.13	-57.84
N6-C7-C9-H14	179.15	-179.12	-179.00
N6-C7-C9-H15	60.46	61.80	62.28
C8-C7-C9-O4	62.75	64.38	64.59
C8-C7-C9-H14	-58.60	-56.61	-56.58
C8-C7-C9-H15	-177.29	-175.68	-175.30
H12-C7-C9-H4	-175.96	-174.37	-174.11
H12-C7-C9-H14	62.68	64.64	64.72
H12-C7-C9-H15	-56.01	-54.43	-54.00
O3-C8-C20-C21	137.76	140.20	141.47
O3-C8-C20-C22	-42.51	-40.47	-39.40
C7-C8-C20-C21	-101.06	-98.65	-97.42
C7-C8-C20-C22	78.66	80.68	81.71
H13-C8-C20-C21	18.82	20.77	21.62
H13-C8-C20-C22	-161.45	-159.91	-159.24
O5-C10-C11-Cl 1	-92.47	-87.81	-89.99
O5-C10-C11-H12	30.82	35.45	33.32
O5-C10-C11-H19	150.79	155.61	153.43
N6-C10-C11-Cl 1	85.22	89.64	87.48
N6-C10-C11-Cl 2	-151.49	-147.10	-149.20
N6-C10-C11-H19	-31.52	-26.94	-29.09
C8-C20-C21-C23	179.05	178.55	178.30
C8-C20-C21-H24	-1.15	-1.80	-2.09
C22-C20-C21-C23	-0.69	-0.78	-0.84
C22-C20-C21-H24	179.11	178.87	178.77
C8-C20-C22-C25	-179.05	-178.64	-178.43
C8-C20-C22-H26	2.18	2.70	2.86
C21-C20-C22-C25	0.68	0.69	0.72
C21-C20-C22-H26	-178.09	-177.97	-177.99
C20-C21-C23-C27	0.15	0.23	0.28
C20-C21C-23-H28	179.76	179.73	179.72
H24-C21-C23-2C7	-179.65	-179.43	-179.33
H24-C21-C23-H28	-0.04	0.07	0.11
C20-C22-C25-C27	-0.14	-0.05	-0.04
C20-C22-C25-H29	-179.85	-179.84	-179.84
H26-C22-C25-C27	178.63	178.60	178.66
H26-C22-C25-H29	-1.08	-1.19	-1.15
C21-C23-C27-C25	0.41	0.43	0.43
C21-C23-C27-N30	-179.99	-179.99	179.96
H28-C23-C27-C25	-179.21	-179.08	-179.03
H28-C23-C27-N30	0.39	0.50	0.50
C22-C25-C27-C23	-0.41	-0.52	-0.54
C22-C25-C27-N30	179.99	179.91	179.92
H29-C25-C27-C23	179.30	179.28	179.27
H29-C25-C27-N30	-0.30	-0.30	-0.27
C23-C27-N30-O31	-179.50	-179.65	-179.52
C23-C27-N30-O32	0.66	0.51	0.64
C25-C27-N30-O31	0.11	-0.06	0.02
C25-2C7-N30-O32	-179.73	-179.90	-179.81

### 4.2 Biological property analysis

**Figure 2:**
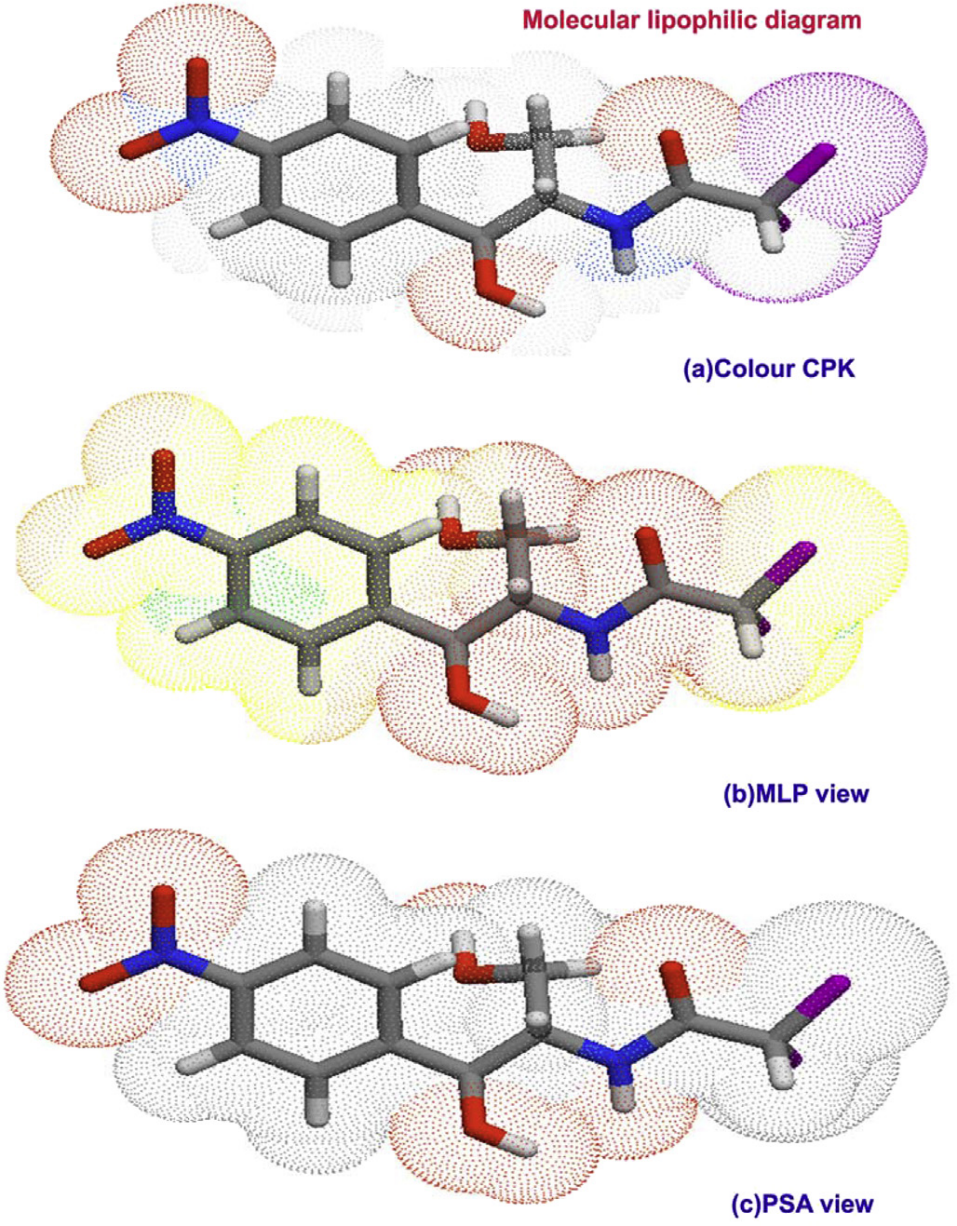
(a) CPK view (b) MLP view (c) PSA view of Chloramphenicol.

The biological parameters were calculated and the Lipophilicity diagrams were drawn and they are listed in Table 2 and related sketches are shown in Figure 2. According to the “Lipinski Five Rule” if the drug molecule has privileged absorption or permeation, the compound should have less than 5 H-bond donors and 10 H-bond acceptors, the molecular weight (MWT) is lesser than 500, Log P (CLogP) is lesser than 5 and the topological surface area of molecule should lesser than 400 A^2^ [8]. But in this case, the HBD and HBA were calculated to be 3 and 5 respectively. Hence, the MWT, LogP and TPSA were measured to be 323.1 g/mol, 0.73, and 115.38A^2^ correspondingly. These values of corresponding rules are obeyed and solubility and permeability of present drug molecule was found to be good and has certain pharmacological activity. The above said observed parameters showed well organized molecular properties and having superior drug's pharmacokinetics; absorption, distribution, metabolism, and excretion.

**Table 2:** Biological property/activity parameters of Chloramphenicol.

Parameters	Values
Hydrogen bond donor count	3
Hydrogen bond acceptor count	5
Rotatable bond count	6
Topological Polar Surface Area	115.38A^2^
Mono isotopic Mass	323.1 g/mol
Exact Mass	323.1 g/mol
Heavy Atom Count	9
Covalently-Bonded Unit Count	1
Log P	0.73
‘N’ ON	7
‘N’OHNH	3
‘N’ violations	0
volume	249.1
GPCR ligand	0.22
Ion channel modulator	0.28
Kinase inhibitor	0.38
Nuclear receptor ligand	0.41
Protease inhibitor	0.21
Enzyme inhibitor	0.11
Drug like score	0.98

Since Covalently-Bonded Unit Count and Heavy Atom Count are reported to be 1 and 9 for this case, the physical property will be limited and accepted. The GPCR ligand of the present drug was calculated to be 0.22 which is very low and it enables membrane domain receptors process was good for signal transduction pathways. The Ion channel modulator value found to be 0.28 and this value of present drug molecule is enough to modulate Ligand-gated ion channels to the ions to travel through membrane. The kinase activity affecting disease and regulate many features which control cell growth and drug compound; inhibit specific kinases are being developed to treat several diseases. For the present case, the Kinase inhibitor was found to be 0.38; though it was low and capable of working for treatment of bacterial infections. The same trend was observed in last work [9].

Protease inhibitor and Enzyme inhibitor are the enzymes which enforce inhibition on pathogenic protozoa and they were found to be 0.21 and 0.11 respectively. They purposively seize replication of a microbial pathogen without troublesome toxicity. The Nuclear receptor ligand was traced to be 0.41 that modulate and regulate promoter-specific regulators of transcription easily and penetrate biological membranes. The bioactive score was calculated with respect to Lipinski “Rule of Five” which was found to be 0.98. This was moderate but it is not enough for the antibiotic compounds to limit the toxicity and side effects. Here, the present drug; chloramphenicol has less than 80% of bio-affinity and it should be improved to use the compound for antibiotic activity.

### 4.3 Mulliken charge gradient analysis

**Figure 3:**
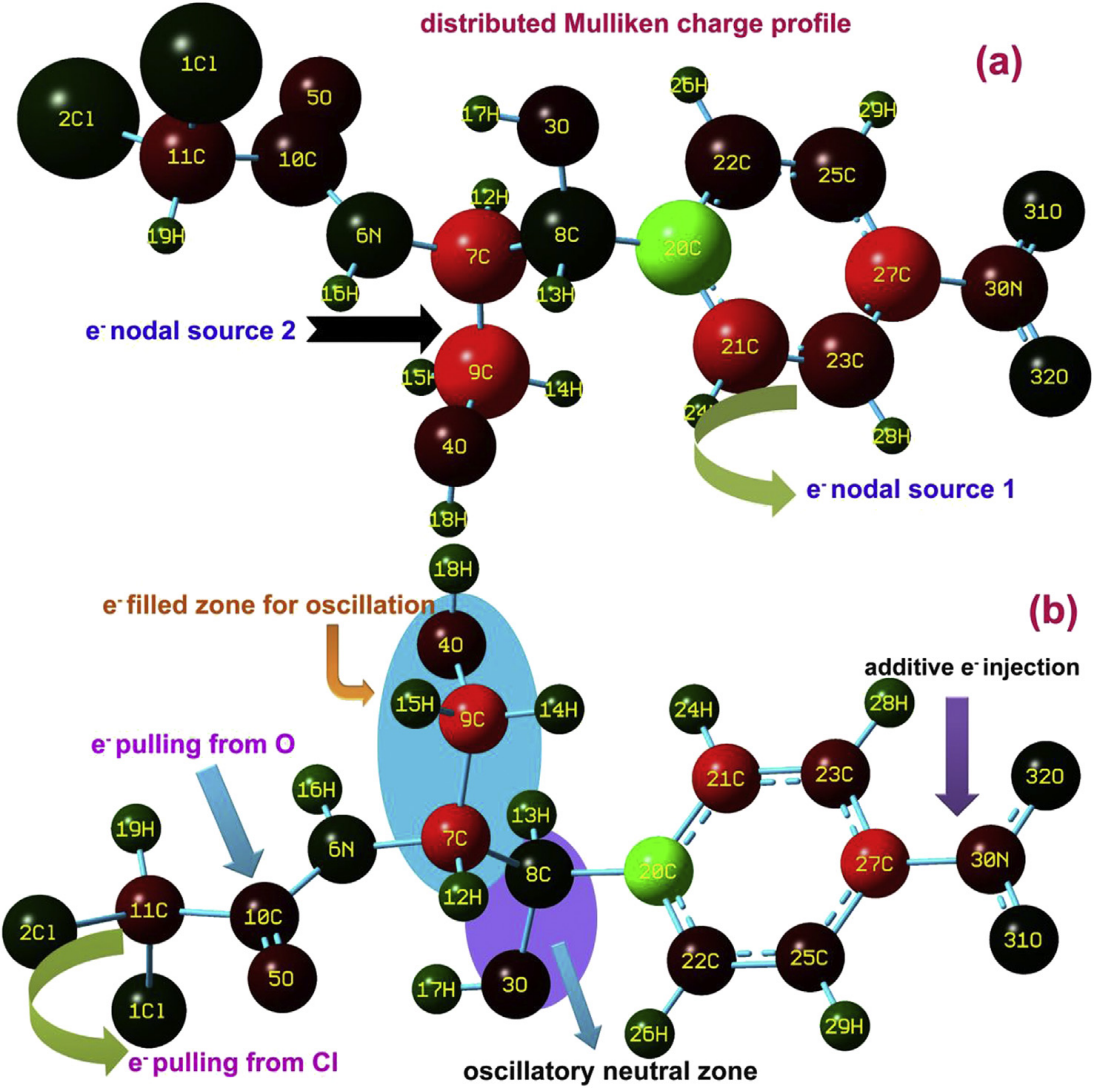
(a) van der Waals radii (b) Bond type Mulliken charge profile of Chloramphenicol.

The molecular orbitals usually organized the occupied and unoccupied energy levels that supporting the electronic transitions offered with respect to the chemical kinetics where in which the structural and chemical property are exposed and such that, the application of the chemical species can be predicted [10]. Such chemical kinetics are usually measured from charge packing assembly among the molecular sites which explicating the charge-domain potential known as chemical potential which causing chemical aided property within the compound [11]. Here the molecular charge depletion diagram was sketched by the hybrid calculations and is depicted in Figure 3.

The title molecular structure has two separate charge domain in which charge collision taking place and they are nitrobenzene and chain of ligand groups. In nitrobenzene domain, the electron sphere around ligand was injected by nitro group and the O of nitro group was appeared to be neutral. In the ring, the charge asymmetrically oscillated, particularly, the negative charges were pulled by C21, C23 and C27 and they were pushed to the chain via C20 (looking at green). Apart from ring, in chain, the charges were pulled from β-hydroxyl group and pushed to the α-methyl hydroxyl group and thereby, the chemical property of first group reacted with second group and adequate chemical potential was supplemented on the chain. But, in chloro-methyl and carbonyl group, the charge alignments were appeared to be confused and negative domain levels was rather depreciated. This idle asymmetric charge movement abolished optimized charge distribution among molecular entities that results static charge assignment and influence on such ligand groups. This condition of charge asymmetricity would boost the molecular property with uncontrolled manner and thus, the molecule behaved as antibiotic with considerable level of toxicity.

### 4.4 Vibrational analysis

#### 4.4.1 Vibrational assignment

**Figure 4:**
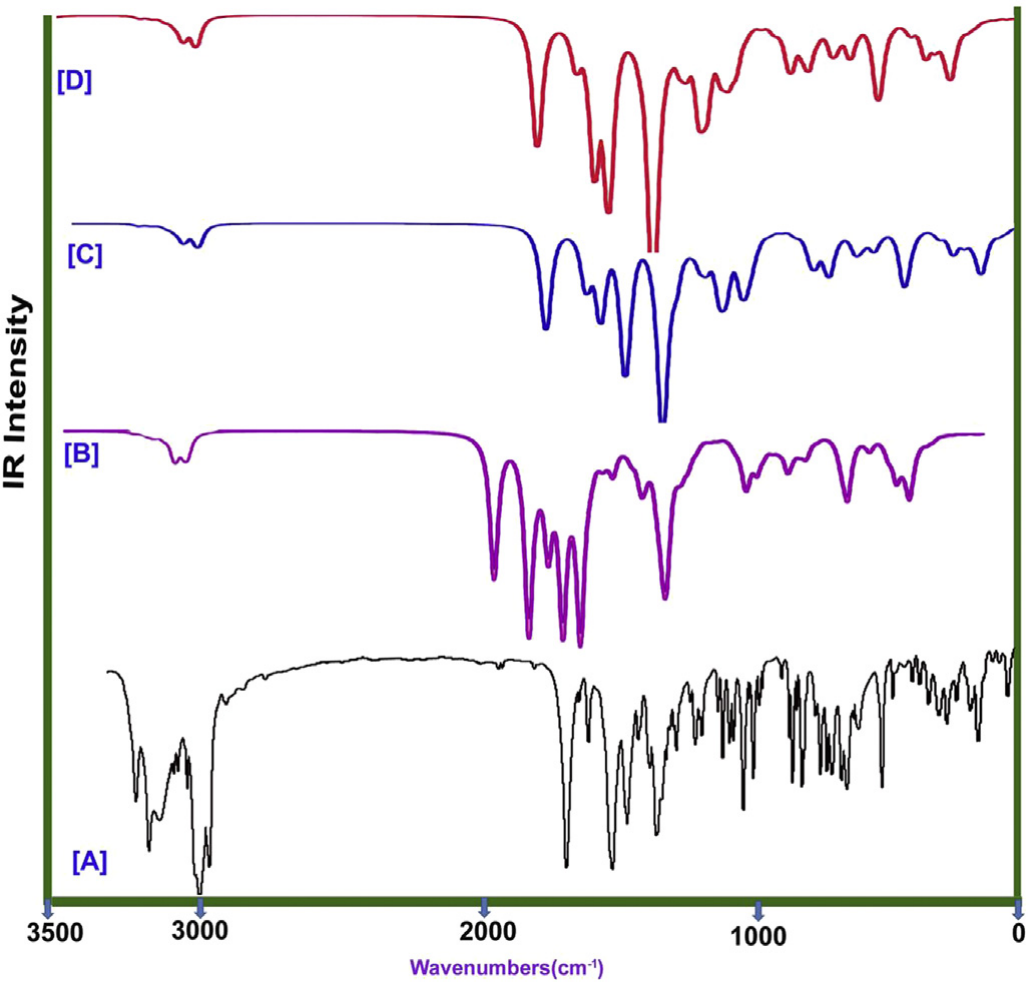
(a) observed (b) HF (c) B3LYP (d) B3PW91 FT-IR spectral pattern of Chloramphenicol.

**Figure 5:**
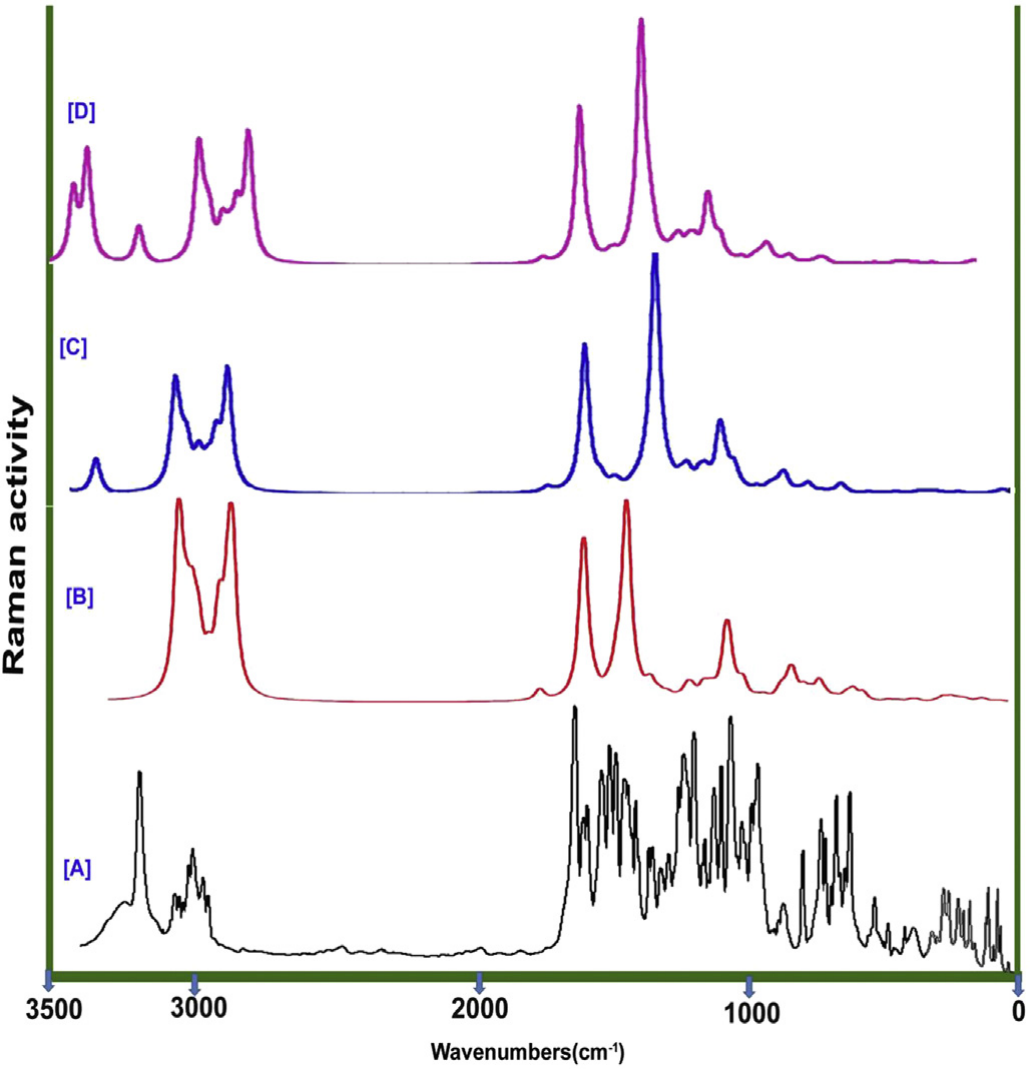
(a) observed (b) HF (c) B3LYP (d) B3PW91 FT-Raman spectral pattern of Chloramphenicol.

The title molecular structure was made up of 32 heavy and lighter atoms and forming homonuclear and heteronuclear bonds by which 90 vibrational modes were calculated. According to the selection rule for cyclic molecule, 32 stretching, 29 in plane bending and 29 out of plane bending vibrations have been observed. The IR and Raman spectral pattern are showed all the active and weak bands and they illustrated in the Figures 4 and 5 in order. The IR and Raman spectral wavenumbers along with the calculated were presented in the Table 3.

**Table 3:** Observed and calculated (HF and DFT) vibrational frequencies of Chloramphenicol.

S. No	Symmetry Species C_S_	Observed frequency (cm^−1^)		Methods		Vibrational Assignments
				B3LYP	B3PW91	
		FT-IR	FT-Raman	6-311++G (d,p)	6-311++G (d,p)	
1	A**′**	3400m	-	3395	3378	(O-H)**υ**
2	A**′**	3380m	-	3388	3371	(O-H)**υ**
3	A**′**	3300vs	3300s	3295	3279	(N-H) **υ**
4	A**′**	3090m	3090m	3096	3081	(C-H) **υ**
5	A**′**	3080w	3080w	3087	3072	(C-H) **υ**
6	A**′**	3020m	-	3018	3003	(C-H) **υ**
7	A**′**	3010m	3010w	3002	2987	(C-H) **υ**
8	A**′**	2950m	2950w	2962	2947	(C-H) **υ**
9	A**′**	2940s	2940w	2950	2935	(C-H) **υ**
10	A**′**	2920s	2920w	2928	2913	(C-H) **υ**
11	A**′**	2880s	2880m	2886	2872	(C-H) **υ**
12	A**′**	2850s	2850w	2856	2842	(C-H) **υ**
13	A**′**	1670vs	1670m	1679	1671	(C=O) **υ**
14	A**′**	1630w	-	1642	1634	(O-H) **δ**
15	A**′**	1600m	1600m	1612	1604	(O-H) **δ**
16	A**′**	-	1580w	1595	1587	(N-H) **δ**
17	A**′**	-	1560w	1567	1559	(C=C) **υ**
18	A**′**	1520vs	-	1532	1524	(C=C) **υ**
19	A**′**	1510vs	1510w	1520	1512	(C=C) **υ**
20	A**′**	1460vs	-	1472	1465	(C-C) **υ**
21	A**′**	1450vs	-	1460	1453	(C-C) **υ**
22	A**′**	1420m	-	1431	1424	(C-C) **υ**
23	A**′**	1370s	1370w	1376	1369	(N-O) **υ**
24	A**′**	1360vs	1360w	1365	1358	(N-O) **υ**
25	A**′**	1340s	1340w	1346	1339	(O-H) **γ**
26	A**′**	1320w	-	1329	1322	(O-H) **γ**
27	A**′**	1280vs	1380w	1289	1283	(C-H) **δ**
28	A**′**	1230m	1230w	1241	1235	(C-H) **δ**
29	A**′**	1210m	-	1222	1216	(C-H) **δ**
30	A**′**	1205m	-	1216	1210	(C-H) **δ**
31	A**′**	1180w	1180w	1191	1185	(C-H) **δ**
32	A**″**	1130w		1128	1122	(C-H) **δ**
33	A**″**	1120s	1120w	1118	1112	(C-H) **δ**
34	A**″**	1090w	-	1087	1082	(C-H) **δ**
35	A**″**	1085w	-	1080	1075	(C-H) **δ**
36	A**″**	1070w	1070s	1065	1060	(N-H) **γ**
37	A**″**	1040vs	1040m	1038	1033	(C-N) **υ**
38	A**″**	1035vs	-	1029	1024	(C-N) **υ**
39	A**″**	995m	995m	1002	997	(C-N) **υ**
40	A**″**	970w	-	1001	996	(C-C) **υ**
41	A**″**	960m	960w	990	985	(C-C) **υ**
42	A**′**	930w	-	954	949	(C-C) **υ**
43	A**′**	900w	900w	921	916	(C-C) **υ**
44	A**′**	895w	-	910	905	(C-O) **υ**
45	A**′**	870m	870w	888	884	(C-O) **υ**
46	A**′**	860vs	860w	871	867	(C-H) **γ**
47	A**′**	855vs	-	865	861	(C-H) **γ**
48	A**′**	840w	840w	854	850	(C-H) **γ**
49	A**′**	820vs	820w	830	826	(C-H) **γ**
50	A**″**	815vs	-	820	816	(C-H) **γ**
51	A**″**	770w	770s	778	774	(C-H) **γ**
52	A**″**	760s	760w	772	768	(C-H) **γ**
53	A**″**	750s	750w	750	746	(C-H) **γ**
54	A**″**	730s	-	728	724	(C-H) **γ**
55	A**″**	710s	710m	701	697	(C-Cl) **υ**
56	A**″**	705s	-	700	697	(C-Cl) **υ**
57	A**″**	670s	670m	698	695	(C-N) **δ**
58		660s	660w	675	672	(C-N) **δ**
59		650s	650w	662	659	(C-N) **δ**
60		620w	-	630	627	(C-C) **δ**
61		610w	610w	627	624	(C-C) **δ**
62		600w	600w	602	599	(C-C) **δ**
63		530s	530w	570	567	(C-C) **δ**
64		525s	-	541	538	(C-O) **δ**
65		480w	480w	492	490	(C-O) **δ**
66		475w	-	487	485	(CCC) **δ**
67		450w	450m	468	466	(CCC) **δ**
68		440w	440m	442	440	(CCC) **δ**
69		410w	410m	421	419	(N-O) **δ**
70		400w	-	398	396	(N-O) **δ**
71		380w	380w	984	979	(C-Cl) δ
72		370w	370w	965	960	(C-Cl) δ
73		350w	350w	948	943	(CCC) **γ**
74		330w	-	328	326	(CCC) **γ**
75		320w	320w	312	310	(CCC) **γ**
76		300w	300w	288	287	(C-N) **γ**
77		280w	280m	274	273	(C-N) **γ**
78		260w	260w	268	267	(C-N) **γ**
79		250w	250w	254	253	(C-C) **γ**
80		210w	210m	222	221	(C-Cl) **γ**
81		200w	200w	198	197	(C-Cl) **γ**
82		190w	-	185	184	(N-O) γ
83		180w	-	175	174	(N-O) γ
84		160w	-	168	167	(C-C) γ
85		150w	150w	159	158	(C-C) γ
86		140w	140w	149	148	(C-C) γ
87		130w	-	128	127	(C-O) γ
88		120w	120w	118	117	(C-O) γ
89		110w	110w	121	120	(C=O) δ
90		100w	100w	110	109	(CHOH) τ

vs – Very strong; s – Strong; m- Medium; w – weak; υ – stretching; α –deformation, δ - In plane bending; γ-out plane bending; τ – Twisting.

#### 4.4.2 Ring vibrations

Even the benzene is substituted by passive and active ligand groups, the ring and allied vibrations are rather affected and it is pronounced by shifting of wavenumbers from higher region to lower region of spectrum. Here, the benzene ring was injected by nitro on one side and chain was another side. Usually, for benzene and its related derivatives, the C-H stretching, in plane and out of plane bending modes assigned in the region 3000-3100 cm^−1^, 1000-1300 cm^−1^ and 710-990 cm^−1^ respectively [12, 13, 14]. Here, the stretching patch was observed in the wavenumbers; 3090, 3080, 3020 and 3010 cm^−1^, the in plane bending modes are observed at 1280, 1230, 1210 and 1205 cm^−1^ and out of plane modes were at 860, 855, 840 and 820 cm^−1^. All the vibrational modes were found at the top end of expected region and they are accepted the spectral boundary of above literatures. From this observation, it was found that, the ring was not infected by lowering of wavenumbers and the ring property was enhanced in the molecular property.

Usually the core and allied CC bonding set up is disturbed by the addition of ligand groups in the benzene ring. The core CC stretching bands are located in the region 1650-1430 cm^−1^ for benzene and its associated derivatives [15, 16, 17]. Here, for this case, the C=C stretching bands were appeared at 1560, 1520 and 1510 cm^−1^ and the C-C stretching modes have been found at 1460, 1450 and 1420 cm^−1^. All those vibrational wavenumbers observed within the characteristics region and they are realigned with respect to their substitutional groups. All the observed bands are placed in discrete manner and they pronounced injected ligand groups. The ring breathing in plane and out of plane vibrations have been observed at 475, 450 and 440 cm^−1^ and 350, 330 and 320 cm^−1^ respectively. Actually, as per the literature [18], they were identified in the region 600-550 cm^−1^ and 500-420 cm^−1^ respectively. All the ring breathing modes are moved down due to the injection of ligand groups and thus the liberation of chemical energy to such group for generating sequential reaction mechanism.

#### 4.4.3 C-Cl vibrations

The halogenic vibrations influence the finger print region of base compound since they are highly electronegative and making high degree of dipole bonds. They influenced physical as well as chemical property due to the high atomic mass and electronic supply capability. For this case, two chlorine atoms were attached with chain; thereby two sets of C-Cl vibrational domains are expected. Usually, the C-Cl stretching, in pane and out of plane bending modes are identified in the region 750-580 cm^−1^ [19], 385-310 cm^−1^ [20] and 280-200 cm^−1^ [21] respectively. Even though, Cl attached with methyl group, stretching, in plane and out of plane bending vibrational bands were found at 710 & 705 cm^−1^, 380 & 370 cm^−1^ and 210 & 200 cm^−1^ respectively. All the vibrational peaks were located at the top end of the expected region which showed the consistency of the halogen bonds that enriched chlorine property on compound.

#### 4.4.4 Nitro group vibrations

The nitro group for this compound was injected over benzene ring and which was represented by their stretching, in plane bending and out of plane bending peaks. They are readily recognized by symmetrical mode of vibrations. They are usually observed in the regions 1320-1390 cm^−1^ for nitrobenzene and its derivatives [22]. Here, two symmetric bands were found at 1370 cm^−1^ and 1360 cm^−1^ for NO_2_ symmetric stretching vibrations. Similarly, peak with very strong to medium intensity is observed in the region 490-400 cm^−1^ and 390-310 cm^−1^ [23] by the vibration of in-plane bending and out-of-plane bending of NO_2_ group. For that, two sets of bands were found at 410 & 400 cm^−1^ and 190 & 180 cm^−1^ for NO_2_ in-plane and out-of-plane vibrations.

#### 4.4.5 C=O vibrations

In general, the C=O stretching vibration is expected in the region 1710-1670 cm^−1^ [24, 25, 26] for carbonyl substituted compounds. Accordingly, the same mode was appeared at 1670 cm^−1^ as very strong band in IR and as medium band in Raman spectra. Due to the presence of C=O in lower band region of characteristics zone, it's part of energy was consumed by amide and α-hydroxyl group. This band should have been observed at the top end of expected zone but this was not happened so. This view showed puzzled atmosphere in the chemical energy distribution on chain and it will generate malfunctioned property in the compound.

#### 4.4.6 N-H and C-N vibrations

Here, the amide group was placed in between carbonyl and hydroxyl groups and due to that, the vibrational bands are expected to be suppressed much. Usually, the N-H stretching, in plane and out of plane bending vibrations for aliphatic primary amines occurred in the wavenumber zone 3400-3300 cm^−1^, 1600-1450 cm^−1^ and 1100-950 cm^−1^ [27, 28] correspondingly. Here, for the same, three bands for stretching, in plane and out of plane bending modes were found at 3300, 1580 and 1070 cm^−1^ in order. Instead of suppression of vibrational bands, the amide vibrations were found to be getting elevated. Since this is not expected in this case, it will make any malfunction on the resultant chemical property in the compound. In this case, the location of C-N bond was related to nitro and amide group and due to which the C-N stretching and in plane and out of plane bending were determined to be broad range and discrete levels in spectrum. Accordingly, they were found at 1040 & 1035 cm^−1^, 670 & 660 cm^−1^ and 300 & 280 cm^−1^ respectively for N-H bond. Similarly, they have assigned at 995, 650 & 260 cm^−1^ respectively for N-O bond. As per the literature [29], all those vibrations were expressed to be rather suppressed and it was happened due to the unequal distribution of chemical potential for Acetamide groups.

#### 4.4.7 C-H vibrations for ethyl and methyl group

The C-H stretching, in plane bending and out of plane bending modes for all groups were found at 2950, 2940, 2920, 2880 & 2850 cm^−1^, 1180, 1130, 1120, 1090 & 1085 cm^−1^ and 815, 770, 760, 730 and 710 cm^−1^ respectively. According to the characteristics region of each and every group of C-H, all the vibrations were observed to be rather moved down to the lower limit of expected region [30].

### 4.5 NMR analysis

**Figure 6:**
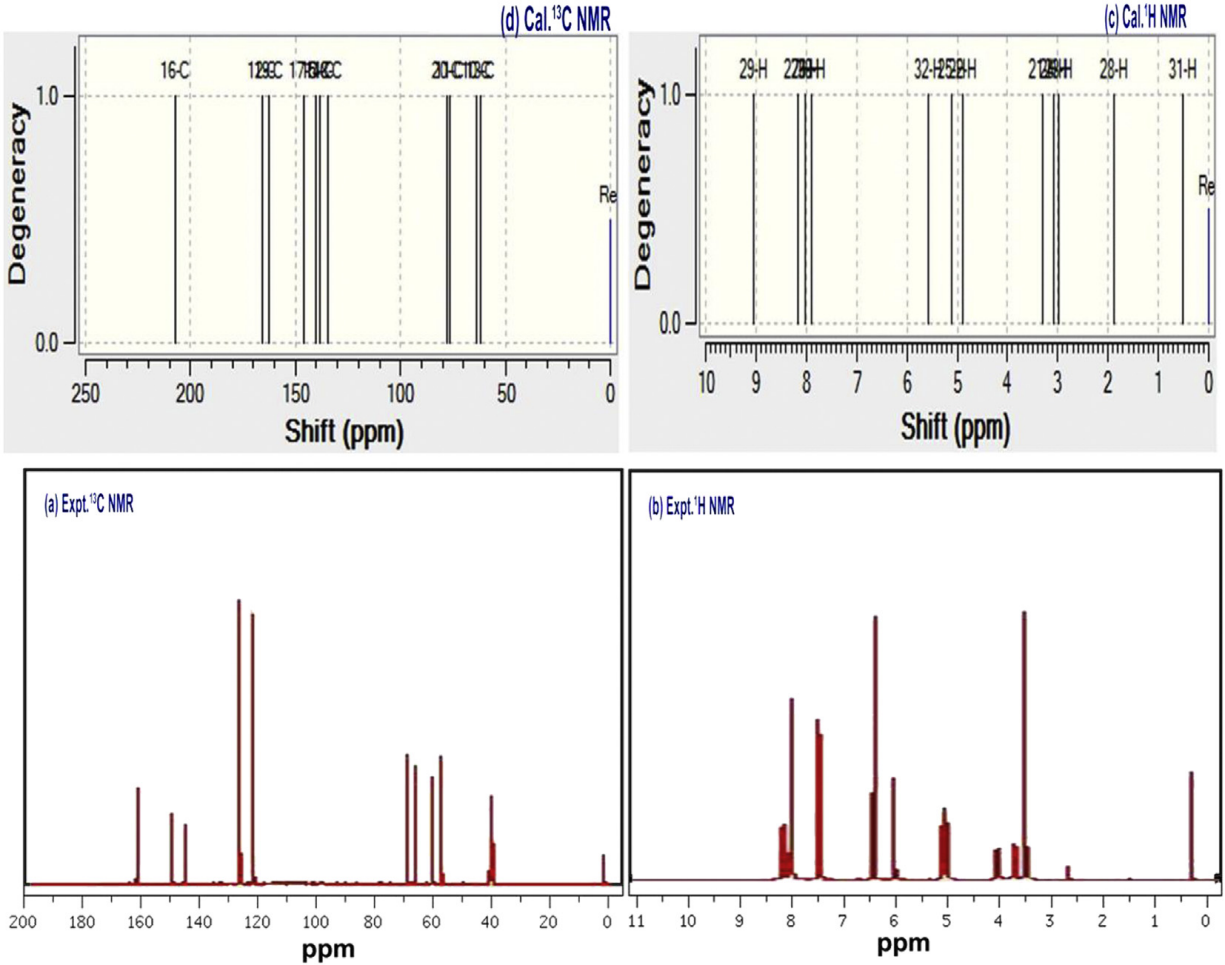
(a) observed^13^C (b) observed ^1^H (c) calculated^13^C (d) calculated ^1^H NMR spectra of Chloramphenicol.

The formation of electron cloud around the carbons is directly influenced by surrounding electronegative and proto-positive atoms. Chemi-dispersive forces existed among the molecular site helped to reorient the electron arrangement apart from the bonding electrons. Not only had the bonding electrons affecting the chemical shift and they are also influenced by the high rated electronegative atoms present around and making deshielding effect as bonded electron. Therefore, the chemical shift path is used to identify the chemical reaction path and its reaction mechanism to induce the chemical effect on molecule. Thus, the assignment of chemical potential is usually replicated in terms of chemical shift of core and allied carbons in the molecule [31]. The chemical shift of all core and allied C and H are depicted in Table 4 and associated spectra are presented in Figure 6.

**Table 4:** Experimental and calculated ^1^H and ^13^C NMR chemical shifts of Chloramphenicol.

Atom position	TMS-B3LYP/6-311++G(2d,p) Shift (ppm)			Experimental shift (ppm)
	Gas	Solvent phase		
		DMSO	CCl_4_	
C7	64.8	65	64.8	66
C8	75.9	76	75.9	60
C9	61.3	61.7	61.5	58
C10	203.5	206.9	204.8	165
C11	77.3	77.5	77.3	69
C20	157.7	160.6	159	161
C21	132.7	135	133.6	128
C22	132.3	148.9	132.5	128
C23	130	131.1	130.3	122
C25	132.6	132.5	132.6	123
C27	154.1	154.6	154.3	162
H12	3.29	3.25	3.2	3.5
H13	4.67	4.88	4.7	6.0
H14	2.81	2.82	2.8	2.8
H15	3.10	3.07	3.09	3.0
H16	4.45	5.14	4.72	5.0
H17	1.48	1.91	1.66	2.0
H18	0.15	0.54	0.16	1.0
H19	5.17	5.57	5.32	6.5
H24	7.64	7.92	7.75	7.6
H26	8.06	8.08	8.09	8.1
H28	8.13	8.24	8.17	8.2
H29	8.35	8.37	8.37	8.3

In benzene ring, C20 and C27 are two substitutional points and are having chemical shift of 160 and 154 ppm respectively. Due to such chemical deshielding, the chemical shift of adjacent carbons (C22 = 148ppm and C21 = 135 ppm) were found to be very high. Similarly, the chemical shift of surrounded carbons; C25 = 132 ppm and C23 = 131 ppm of C27 = 154ppm. The chemical shift was extended to the adjoining carbons in which, the chemical potential of substitutional groups were penetrated through the core carbons of ring by pushing electron domain and it was limited up to certain carbons of ring. Hence, the chemical property was operated with respect to the blending of chemical potential of substituted groups.

In the case of chain, the chemical shift of C7, C8, C9 and C11 were found to be 65, 76, 61 and 77 ppm respectively and these points of C were indentified to aliphatic. In those nodal carbons, though, different ligand groups with different mass are injected in corresponding carbons, they belong to lower field of chemical shift. This view showed the random motion of electron cloud in certainty condition which explained the ambuscade atmosphere of antibiotic activity and due to such condition, sometimes the drug activity will be randomly opened and uncontrolled manner. In the case of chemical shift of H of ring, all the H was having moderate shift and which were found to be directly influenced by core shift.

### 4.6 Frontier molecular interaction analysis

**Figure 7:**
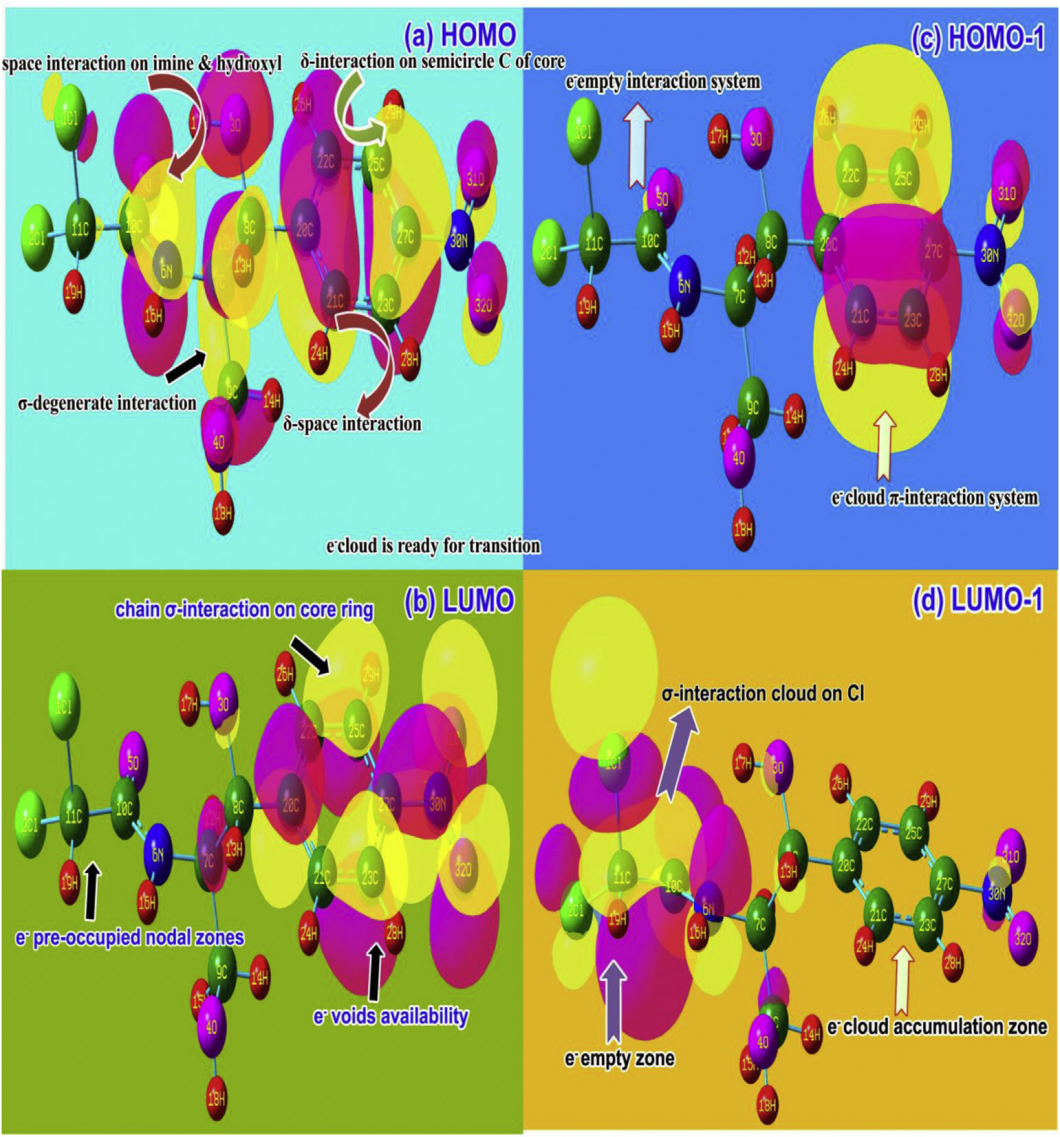
(a) HOMO (b) LUMO (c) HOMO-1 (d) LUMO-1FMO representation of Chloramphenicol.

The formation of molecular orbitals in hybrid process is enforced by the suitable combination of LCAO in the molecule in which two sets of occupied and unoccupied energy systems are fashioned. The transitions in terms of electronic energy is stimulating chemical property that are represented by the partial involvement of substitutional groups on the base species. The orbital overlapping s usually taking place by the interaction of orbitals between two or many energy systems and it produced degenerate energy domains in which the hybrid rational chemical potential is generated. Such potential is used to recognize the drug property along with toxicity. The frontier modified interaction system for present case is illustrated in Figure 7.

In this frontier profile, the HOMO was appeared around benzene ring, hydroxyl group and Acetamide group in which δ-interaction bonding system was found on semicircle of ring. The σ-bonding overlapping system was found around carbonyl and hydroxyl group and special type space interaction was determined on C=O and hydroxyl group in which the excited electron cloud ready to transit to unoccupied energy system. So total available electron cloud looking at group except chloro-methyl group. Particularly, two α and β-hydroxyl group were blended with one another and ready to exchange hybrid electron cloud.

In LUMO system, σ-bonding system was acknowledged on C of benzene ring and space orbital interaction of nitro group also participated in the LUMO domain in which the energy system was found to be ready to receive electronic energy to produce drug mechanism. The chain; carbonyl, hydroxyl group and methyl-chloro groups was occupied by pre-electron cloud due to which no chemical energy was transferred to chain. But it never to be happened to regulate the drug activity. In this condition, the uncompleted ligand ratio on drug process induced such that they provide insufficient drug activity. In HOMO-1, the orbital interaction was concentrated on benzene ring only and in LUMO-1, σ-blowing orbitals were concentrated only on amide and chloro-methyl group and other groups were pre-occupied.

### 4.7 CT complex analysis in UV-Visible spectra

**Figure 8:**
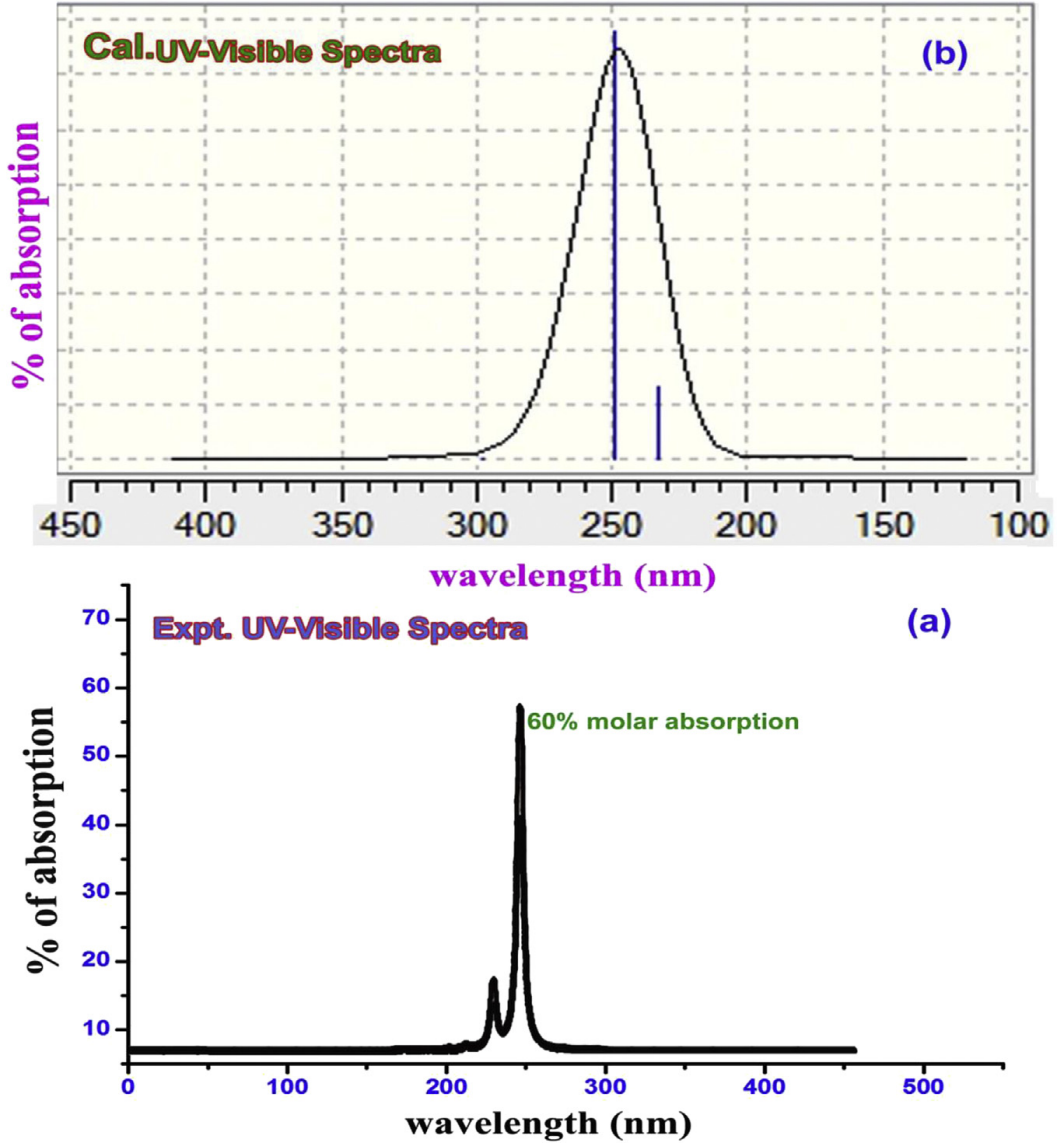
(a) observed (b) calculated UV-Visible spectra of Chloramphenicol.

The amalgamation of electronic energy levels in the molecular orbitals is collective of vibrational energy systems in which chemical potential is generated by viable transitions among space and degenerate interaction region in molecular site [32]. Even after the molecular orbital arranged the molecule itself, the important electronic exchange domain known as charge transfer complex (CT complex) is generally formed by which the entire drug mechanism is controlled and operated. It can be found by identifying transitions located zone that is addressed by individual transition assigned in the UV-Visible spectral parameter Table 5 and the diagram is displayed in Figure 8. Three sets of transitions were recognized when n = 3 in calculation process and were assigned at 365, 295 and 290 nm with the chemical energy gap of 3.39, 4.20 and 4.26 eV fitted with oscillator strength of 0.001, 0.13 and 0.004 respectively. These transitions was HOMO to LUMO with 63% contribution and assigned to n→π∗ system and located in Quartz UV region which was represented by R-Band (German, radikalartig).

**Table 5:** Theoretical electronic absorption parameters of Chloramphenicol.

λ (nm)	E (eV)	(f)	Transition Level	Major contribution	Assignment	Region	Bands
Gas							
365.08	3.3960	0.001	H+1→L (63%)	H→L (63%)	n→π∗	Quartz UV	R-band (German, radikalartig)
295.16	4.2005	0.0134	H→L (68%)				
290.76	4.2642	0.0041	H+1→L(53%)				
DMSO							
349.41	3.5483	0.0000	H→L (67%)	H→L (68%)	n→π∗	Quartz UV	R-band (German, radikalartig)
325.20	3.8126	0.0193	H+1→L (67%)				
313.37	3.9564	0.2047	H+1→L (67%)				
CCl4							
357.87	3.4645	0.0000	H+2→L (69%)	H→L (68%)	n→π∗	Quartz UV	R-band (German, radikalartig)
308.59	4.0178	0.0201	H+1→L(68%)				
4.1519	4.1519	0.2573	H→L (68%)

All the assignment of electronic levels demonstrated to be doubly degenerate levels and was located at 230 and 250 nm and it was verified and validated by experimental spectra. In calculated spectra, all the energy levels were verified with two solvent phases; DMSO and CCl_4_. According to the spectral values assigned degenerate interaction lobe system, the identified CT complex was found to be C=N and C=C bonds. These two bonds acted as main resource to produce antibiotic and antibacterial activity. In same spectra, some wavenumber nodes were present which showed that, the additional property generated in the compound and this may be additive property or drug break down which to be identified and removed by calibrating structure activity.

### 4.8 Physico-chemical properties

The fine and tuned molecular structure is usually identified by optimizing energy which was calculated to be -1832 in both region of IR and UV-Visible, showed very precise optimization of molecule. The electron affinity of compound revealed the electron cloud ability to process the chemical potential to produce accurate chemical property and here it was observed to be 7.73 and 7.89 eV for the present case which confirmed that the drug ability of the compound [33]. The ionization potential is used to measure the property consistency of the compound and it was measured to be 2.96 and 3.08 eV respectively. This is moderate chemical energy which explained the property stability of the compound. The chemical energy gap showed the drug permanence and chemical rigidity to react with other molecule which usually made malfunction that can be measured from the observation of energy gap of 4.77 and 4.81 eV in IR and UV-Visible region. From this value, it was concluded that, the molecule was represented by better drug stability and it would not be react with nearby molecule easily. The physico-chemical parameters are listed in Table 6.

**Table 6:** Calculated energies, chemical hardness, electro negativity, Chemical potential, Electrophilicity index of Chloramphenicol in UV-Visible region.

Parameter	B3LYP 6311++G (d,p)	UV-Visible	Electrophilicity charge transfer (ECT) (ΔN_max_)_A_-(ΔN_max_)_B_
E_total_ (Hartree)	-1832.63	-1832.47	
E_HOMO_ (eV)	7.73	7.89	
E_LUMO_ (eV)	2.96	3.08	
ΔE_HOMO-LUMO gap_ (eV)	4.77	4.81	
E_HOMO-1_ (eV)	7.99	7.98	-1.123
E_LUMO+1_ (eV)	2.96	3.08	
ΔE_HOMO-1-LUMO+1 gap_ (eV)	5.03	4.90	
Chemical hardness (η)	2.38	2.40	
Electronegativity (χ)	5.34	5.48	
Chemical potential (μ)	5.34	5.48	
Chemical softness(S)	-9.54	-9.62	
Electrophilicity index (ω)	5.98	6.26	
Dipole moment	6.88	6.32	
ETC	3.12	3.25	

The chemical hardness for the organic compound is important drug feature which directly evaluate subsequent drug process and it is time dependent. It was determined to be 2.38 and 2.40 eV for both region which explicit that, the drug process was stabilized in the present case in both region. The chemical softness is also important structural and drug property which notified easy drug process and alternation or customization capability of chemical compound. This was determined to be -9.54 and -9.62 in both region which was observed to be very high and the present compound can be customized to add some more drug functions. The Electronegativity and Electrophilicity index are electron gradient parameters and they were found as 5.34 & 5.48 eV and 5.98 & 6.26 eV respectively. These values proved electron cloud process was taking place and parametric oscillation in good order and it enable drug tolerance. The electrophilicity charge transfer is showed the electron cloud motion or movement which results drug mechanism. The path direction is always from ligand to base compound, but in this case it was calculated to be -1.123 which showed negative sign and the path direction was nonconventional and it should be altered and the direction to be changed to enhance sufficient drug activity.

### 4.9 Biological characteristics

The charge polarization in first order and second order in the molecular site is manifested by the observation of first order polarizability and hyperpolarizability. The polarization of charges due to the existence of dispersion forces in different planes at molecular site used to measure the hyperactive pressure which helped to evaluate biological affinity of the compound [34]. Here, all the parameters are presented in Table 7 in which the biological parameters are illustrated in plane and coordinate vice.

**Table 7:** The dipole moments μ (D), the polarizability α(a.u.), the average polarizability α_o_ (esu), the anisotropy of the polarizability Δα (esu), and the first hyperpolarizability β(esu) of Chloramphenicol.

Parameter	a.u.
α_xx_	-181.22
α_xy_	4.42
α_yy_	-121.93
α_xz_	-1.24
α_yz_	2.20
α_zz_	-134.35
α_tot_	266.18
Δα	327.15
μ_x_	-3.48
μ_y_	-5.72
μ_z_	1.58
μ	6.88
β_xxx_	-319.94
β_xxy_	-48.36
β_xyy_	-5.81
β_yyy_	-46.40
β_xxz_	61.75
β_xyz_	-4.41
β_yyz_	-6.09
β_xzz_	-1.91
β_yzz_	-10.84
β_zzz_	0.784
β_tot_	1295

The polarizability was measured in α_xx_, α_yy_ and α_zz_ planes which are to be -181.22, -121.93 and -134.35 respectively and in all planes there were considerable amount of polarization coefficient observed that is the good indicator to notify the polar sequence of molecular arrangement results good biological activity. The total polarizability (α_tot_) and average polarizability (Δα) were determined to be 266.18 X10^−33^ esu and 327 X10^−33^ esu respectively. These are collectively measured the molecule as a whole which clear that, the biological activity was induced in good order. The hyperpolarizability usually measures hyperactive molecular arrangement for developing biological affinity and ability which was calculated to be 1295 X10^−33^ esu that showed hyperactive mechanism for biological liberation of the molecule.

### 4.10 MEP profile analysis

**Figure 9:**
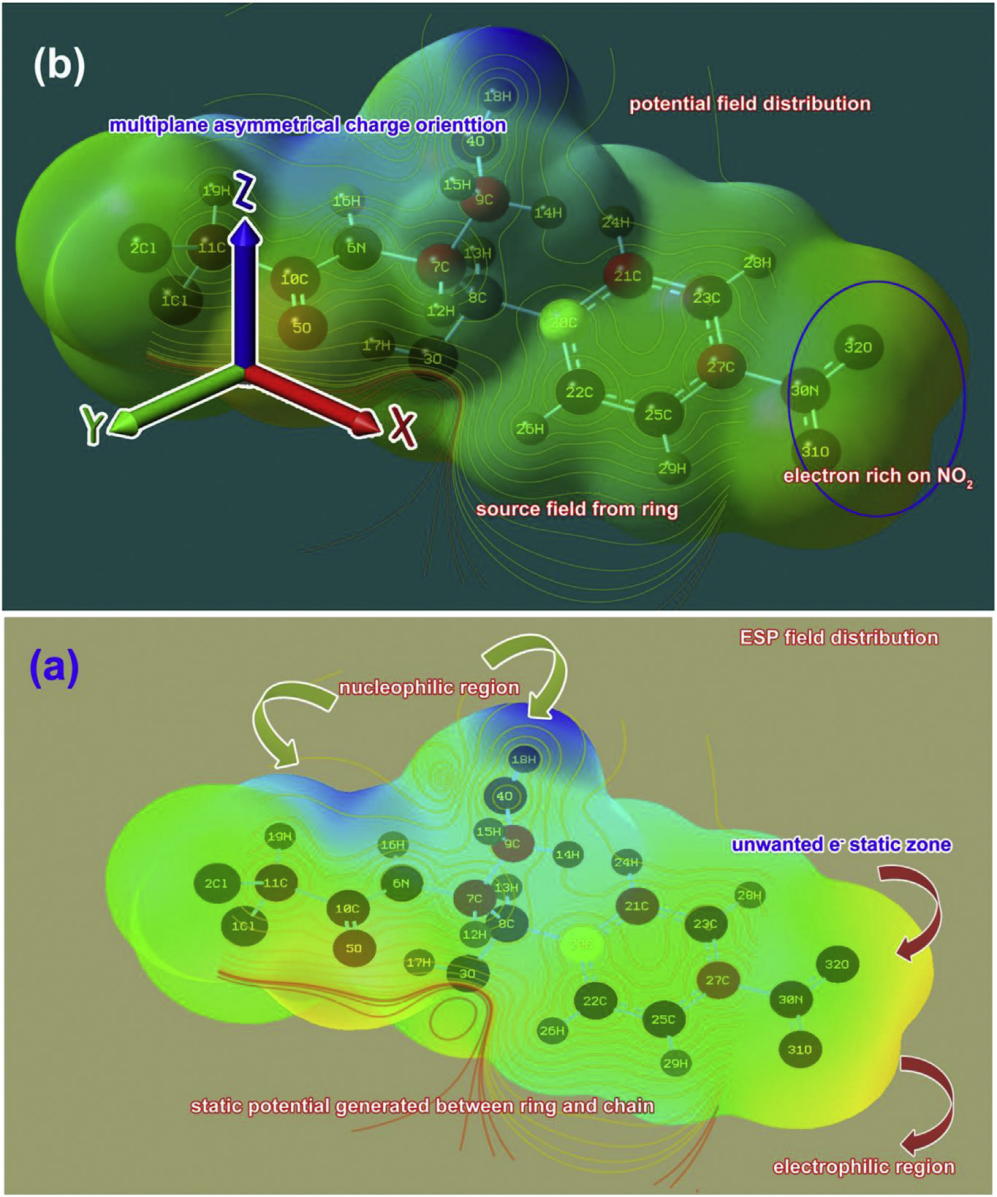
(a)View 1, (b) View 2 MEP sketch of Chloramphenicol.

The polarizability is also measured by the charge depletion gradient over the molecule due to the rational repulsive force between polar and non polar entities. In this molecular charge progression, two separate regions are produced and they are nucleophilic and electrophilic zones. These separate charge incline are differentiated in three different coordinates or internal coordinates of molecule which clearly displayed the molecular static potential field and concentration electronic domain potential to illustrate physical and chemical property. The MEP field distribution of present molecule is portrayed in Figure 9 and the color gradient from blue region to red region represents protonic and electronic rich region respectively.

In this case, the potential field was used to categorize two opposite potential and here the electrophilic region looking at NO_2_ and C=O groups where the electronic tensor gradient was observed more than rest of the other region. The nucleophilic region was appeared over amide and β-hydroxyl group, where the protonic content is concentrated energetically. Other rest areas are acknowledged the intermediate region and this field of distortion of neutral charge zone resulting asymmetry of charge domain. The protonic area was appeared in one plane only while electronic content was amalgamated on two dimensions since the concern groups were placed in two planes. Form this observation inferred that, two opposite charge depletion made obstruction between two regions and causing consistent molecular dipole. Apart from that, at α-hydroxyl group showed another electrophilic region which denote perplex of charge depletion that reports the unwanted property induced in the compound.

### 4.11 NBMO analysis

The molecular chemi-potential is usually inhabited on energy transitions taking place among nonbonding energy system of bonding domains in molecular site. The potential of such transitions can be measured from the energy calculations and the detailed energy transitions are given in Table 8. The number of transitions is depending upon the number of degenerate systems that formed during the molecular orbital amalgamation. Here, some of important transitions with huge amount of energy insist the prepared drug mechanism in chemical reaction such as drug activity is depicted.

**Table 8:** The calculated NBMO of Chloramphenicol by second order Perturbation theory.

Donor (i)	Type of bond	Occupancy	Acceptor (j)	Type of bond	E2 kcal/mol	Ej – Ei au	F(I j) au
**C20-C21**	π		**C23-C27**	π∗	23.69	0.28	0.072
**C21-C23**	σ		**C27-N30**	σ ∗	4.22	0.99	0.059
**C21-H24**	σ	1.97836	**C20-C22**	σ ∗	4.76	1.09	0.064
**C22-C25**	π		**C27-N30**	π∗	4.23	0.98	0.059
**C22-C25**	π		**C20-C21**	π∗	22.46	0.28	0.072
**C22-C25**	π	1.97446	**C23-C27**	π∗	20.85	0.27	0.068
**C22-H26**	σ	1.97757	**C20-C21**	σ ∗	4.82	1.08	0.064
**C22-H26**	σ		**C25-C27**	σ ∗	3.54	1.07	0.055
**C23-C27**	π	1.97544	**C25-C27**	π∗	4.77	1.28	0.070
**C23-C27**	π		**C22-C25**	π∗	20.08	0.30	0.070
**C23-C27**	π		**N30-O31**	π∗	27.54	0.14	0.060
**N30-O31**	**π**	1.99575	**O32**	**π∗**	12.43	0.18	0.079
**N30-O31**	**π**		**C23-C27**	**π∗**	4.07	0.46	0.043
**N30-O31**	**π**		**N30-O31**	**π∗**	7.54	0.32	0.052
**O5**	LP	1.99974	**C10**	N∗	19.74	0.81	0.332
**O32**	LP	1.99979	**N30**	N∗	20.04	0.86	0.214
**Cl 2**	LP		**Cl 1-C11**	σ ∗	10.08	0.40	0.056
**O3**	LP	0.00243	**C7-C8**	σ ∗	4.90	0.64	0.050
**O3**	LP		**C8-H13**	σ ∗	7.59	0.68	0.064
**O4**	LP	0.00189	**C9-H14**	σ ∗	6.59	0.70	**0.061**
**O4**	LP		**C9-H15**	σ ∗	5.26	0.70	0.054
**O5**	LP	0.00283	**C10**	N∗	17.00	1.59	0.147
**O5**	LP		**N6-C10**	**π∗**	25.34	0.70	0.121
**O5**	LP	0.00283	**C10-C11**	σ ∗	24.89	0.57	0.107
**N6**	LP	0.00749	**O5-C10**	π∗	57.55	0.29	0.116
**O31**	LP		**C27-N30**	σ ∗	12.33	0.56	0.074
**O31**	LP		**N30-O32**	**π∗**	19.02	0.72	0.106
**O32**	LP	0.00302	**N30**	N∗	5.90	1.90	0.095
**O32**	LP		**C27-N30**	σ ∗	4.17	1.07	0.061
**O32**	LP		**C27-N30**	σ ∗	12.27	0.56	0.074
**O32**	LP		**N30-O31**	σ ∗	18.91	0.73	0.106
**O32**	LP		**N30**	N∗	3.44	2.39	0.095
**O32**	LP		**N30-O31**	**π∗**	163.08	0.14	0.139
**O5-C10**	π	0.01605	**Cl 1-C11**	σ ∗	9.81	0.06	0.053
**C10-C11**	σ		**N6-C7**	σ ∗	4.71	0.02	0.034
**C23-C27**	π	0.02245	**C23**	π∗	3.85	0.37	0.077
**C23-C27**	π		**C20-C21**	π∗	284.33	0.01	0.084
**C23-C27**	π		**C22-C25**	π∗	217.93	0.01	0.081
**N30-O31**	**π**	0.05452	**O31**	π∗	3.48	0.97	0.093
**N30-O31**	**π**		**C23-C27**	π∗	14.59	0.14	0.058

In ring, first transition was identified in π- π∗ bonding system (occupation energy = 1.97), represented by the energy of 23.69 kcal./mol between C20-C21 and C23-C27. Similarly, another transition from C22-C25 to C20-C21 and C23-C27 with consumption of energy of 22.46 and 20.85 kcal./mol at π- π∗ bonding system. In same way, from C23-C27 to C22-C25 and N30-O31, the transitions were assigned on π- π∗ system with absorbed energy; 20.08 and 27.54 kcal./mol respectively. In lone pair system, two transitions from O5 to C10 and from O32 to N30 were determined with utilizing energy of 19.74 and 20.04 kcal./mol. another two transitions were observed from O5 to N6-C10 and C10-C11 and they were symbolized by LP- π∗ and σ ∗ respectively. In amide group, from N6 to O5-C10, characterized by π- π∗ system by taking energy of 57.55 kcal./mol. In NO_2_ group, the collective transitions from O32 to N30-O31 bonding system were observed and they measured to be 163.08 kcal./mol. this is huge amount of energy which described the major role in participating resultant property of the compound. Another well pronounced transitions from C23-C27 to C20-C21 and C22-C25, were located in ring which was measured to be 284.33 and 217.93 kcal./mol.

From these transitions, it was clear that; considerable amount of chemical potential was transferred among the core carbons of the nitrobenzene ring and α-hydroxyl and β-hybrid hydroxyl groups whereas there was no significant amount of energy transferred among the Acetamide bonding system and chloromethyl groups. As per the property induced in the present molecular system, extensive amount of energy should have been observed on left moiety of molecular structure. But in this case, this was not happened so. This is the major drawback in the preparation of chemical property. Hence, due to this reason, the malfunction of drug property may be taking place and that to be removed for the molecule for being successful drug.

### 4.12 VCD characterization

**Figure 10:**
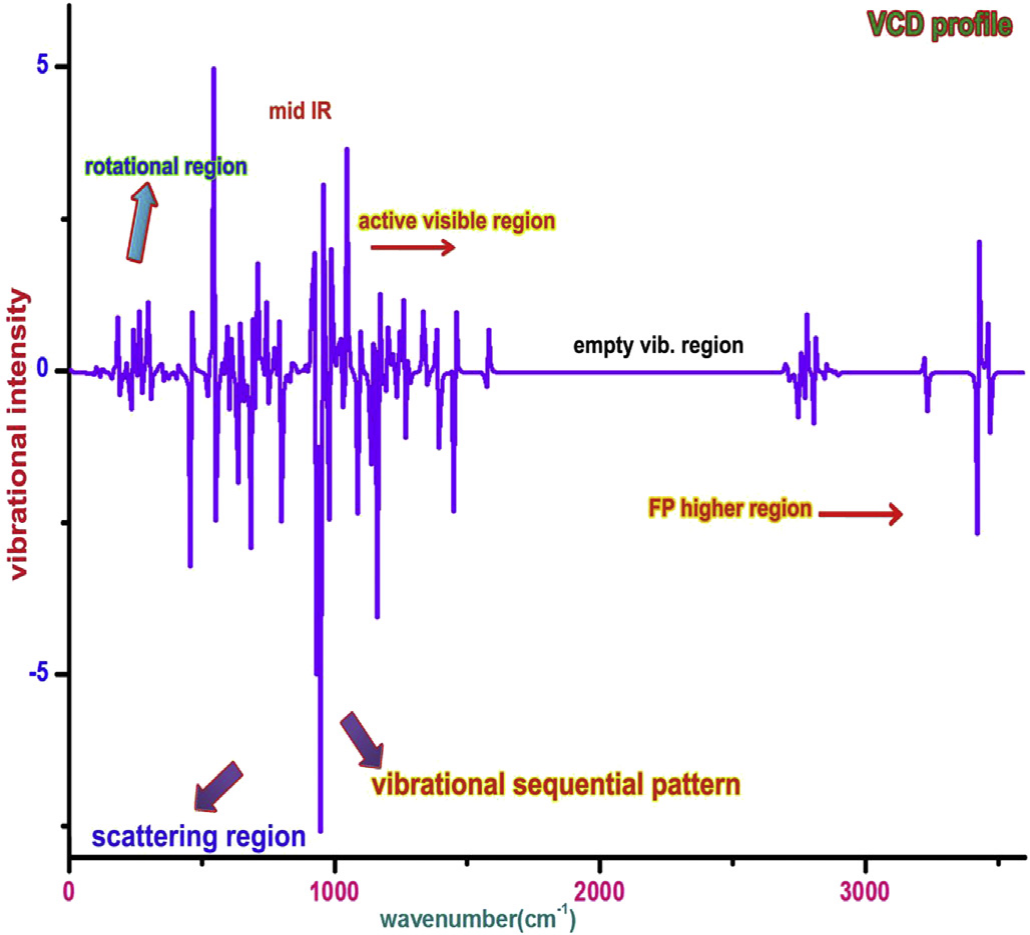
VCD spectra of Chloramphenicol.

The VCD of the organic molecule is used to study the toxicity and unwanted function over the property. The VCD for this case is displayed in Figure 10 in which the characteristics of vibrations in transmission and absorption modes are presented. In rotational region of spectrum there were some unequal peaks observed which represents that, in this region, some molecular elements are found to be react not good manner. This may be breathing region of Acetamide group. In mid-IR region, the ratio between absorption and transmission was rather disturbed and it may be along with the Acetamide group. In finger print region known as near IR region, the peaks were observed with balanced mode which revealed the characteristics region of other elements of the molecule. In such region, the peaks were found to be established in good order and being notified the drug characteristics of the compound.

## 5 Conclusion

In this work, in order to expose biological and drug ability of present compound; Chloramphenicol, the structural and physico-chemical properties were interpreted using the molecular spectroscopic tool; FT-IR, FT-Raman, NMR and UV-Visible, along with the theoretical method of calculation. All the supportive analyses have been performed to evaluate the drug properties and the malfunction of drug process known as toxicity was identified and method of reduction was notified. The discussion was made on obtained results using different analyses upon which some useful conclusions were drawn and are followed;

• The bond angles were distorted in ambiguity form which denoted that, the substitutional groups were not attached and grouped randomly. This atmosphere in molecular site should produce strange chemical property in the compound and the toxicity is induced above the expected limit.
• The bioactive score was calculated with respect to Lipinski “Rule of Five” which was found to be 0.98. This was moderate but it is not enough for the antibiotic compounds to limit the toxicity and side effects. Here, the present drug; chloramphenicol has less than 80% of bio-affinity and it should be improved to use the compound for antibiotic activity.
• In chloro-methyl and carbonyl group, the charge alignments were appeared to be confused and negative domain levels was rather depreciated. This idle asymmetric charge movement abolished optimized charge distribution among atomic entities which results static charge assignment on such ligand groups. This condition of charge asymmetricity usually boosting the molecular property with uncontrolled manner and thus, the molecule behaved as antibiotic with considerable level of toxicity.
• The nitro, halogen, ring and hydroxyl group vibrations were observed in good order whereas Acetamide and its surrounding bonded system have affected much and produces proportionate break down of drug process mechanism. Thus, the toxicity was induced further well above the expected limit.
• From frontier molecular interaction, it was obtained that, the chain; carbonyl, hydroxyl group and methyl-chloro groups was occupied by pre-electron cloud due to which no chemical energy was transferred to chain. But it should not be happened to regulate the drug activity. In this condition, the unsaturated drug process induced which will not provide sufficient drug activity.
• The CT complex analysis reveals that, according to the spectral values assigned degenerate interaction lobe system, the identified CT complex was found to be C=N and C=C bonds. These two bonds acted as main resource to produce antibiotic and antibacterial activity. In same spectra, some wavenumber nodes were present which showed that, the additional property generated in the compound and this may be additive property or drug break down which to be identified and removed by calibrating structure activity.
• The electrophilicity charge transfer is showed the electron cloud motion or movement which results drug mechanism. The path direction is always from ligand to base compound, but in this case it was calculated to be -1.123 which showed negative sign and the path direction was nonconventional and it should be altered and the direction to be changed to enhance sufficient drug activity.
• From NBMO analysis, it was observed that, as per the property induced in the present molecular system, extensive amount of energy should have been observed on left moiety of molecular structure. But in this case, this was not happened so. This is the major drawback in the preparation of chemical property. Hence, due to this reason, the malfunction of drug property may be taking place and that to be removed for the molecule for being successful drug.

## Declarations

### Author contribution statement

S Ramalingam: Contributed reagents, materials, analysis tools or data.

A Sathya: Performed the experiments; Analyzed and interpreted the data; Wrote the paper.

T Prabhu: Conceived and designed the experiments.

### Funding statement

This research did not receive any specific grant from funding agencies in the public, commercial, or not-for-profit sectors.

### Competing interest statement

The authors declare no conflict of interest.

### Additional information

No additional information is available for this paper.
